# Study on the Interrater Reliability of an OSPE (Objective Structured Practical Examination) – Subject to the Evaluation Mode in the Phantom Course of Operative Dentistry

**DOI:** 10.3205/zma001060

**Published:** 2016-08-15

**Authors:** Laura Schmitt, Andreas Möltner, Stefan Rüttermann, Susanne Gerhardt-Szép

**Affiliations:** 1 Goethe-University Frankfurt am Main, Carolinum Dental University Institute GmbH, Department of Orthodontics, Frankfurt/Main, Germany; 2 University Heidelberg, Medical Faculty, Competence Centre for Examinations in Medicine/Baden-Württemberg, Heidelberg, Germany; 3 Goethe-University Frankfurt am Main, Carolinum Dental University Institute GmbH, Department of Operative Dentistry, Frankfurt/Main, Germany

**Keywords:** OSCE, OSPE, checklist, evaluator, instructor's manual, feedback, dentistry

## Abstract

**Introduction: **The aim of the study presented here was to evaluate the reliability of an OSPE end-of-semester exam in the phantom course for operative dentistry in Frankfurt am Main taking into consideration different modes of evaluation (examiner’s checklist versus instructor’s manual) and number of examiners (three versus four).

**Methods: **In an historic, monocentric, comparative study, two different methods of evaluation were examined in a real end-of-semester setting held in OSPE form (Group I: exclusive use of an examiner’s checklist versus Group II: use of an examiner’s checklist including an instructor’s manual). For the analysis of interrater reliability, the generalisability theory was applied that contains a generalisation of the concept of internal consistency (Cronbach’s alpha).

**Results: **The results show that the exclusive use of the examiner’s checklist led to higher interrater reliability values than the in-depth instructor’s manual used in addition to the list.

**Conclusion:** In summary it can be said that the examiner’s checklists used in the present study, without the instructor’s manual, resulted in the highest interrater reliability in combination with three evaluators within the context of the completed OSPE.

## Zusammenfassung

**Einleitung: **Ziel der vorliegenden Studie war es, die Reliabilität einer OSPE-Semesterabschlussprüfung im Phantomkurs der Zahnerhaltungskunde in Frankfurt am Main unter Berücksichtigung unterschiedlicher Bewertungsmodi (Prüfer-Checkliste versus Dozentenmanual) und PrüferInnenanzahl (drei versus vier) zu evaluieren.

**Methoden:** Im Rahmen einer historischen monozentrischen Vergleichsstudie wurden zwei verschiedene Bewertungsmodi (Gruppe I: Verwendung ausschließlich einer Prüfer-Checkliste versus Gruppe II: Verwendung einer Prüfer-Checkliste inklusive eines Dozentenmanuals) im Rahmen einer realen Semesterabschlussprüfung, die in OSPE-Form abgehalten wurde, evaluiert. Zur Analyse der Interrater-Reliabilität wurde die Generalisierbarkeitstheorie verwendet, die eine Verallgemeinerung des Konzepts der internen Konsistenz (Cronbachs alpha) beinhaltet.

**Ergebnisse: **Die Ergebnisse zeigen, dass die alleinige Verwendung der Prüfer-Checkliste zu höheren Interrater-Reliabilitätswerten führte als das zusätzlich zu der Liste verwendete ausführliche Dozentenmanual.

**Schlussfolgerung: **Zusammenfassend kann festgehalten werden, dass die in der vorliegenden Studie verwendete Prüfer-Checkliste ohne Dozentenmanual im Rahmen der durchgeführten OSPE die höchste Interrater-Reliabilität ergab in Kombination mit der Anzahl von drei BewerterInnen.

## Introduction and Problem Definition

Performance checks constitute a central element of teaching; their evaluation is characterised primarily by the quality criteria of objectivity, reliability and validity [1], [2]. A GMA (Society for Medical Education) guideline [1] existing for this purpose and the basic standards of the WFME (World Federation for Medical Examination) [3] indicate the following criteria:

- the examinations must be justiciable
- the examination procedure is based upon learning goals and the learning effect on students
- the examination procedures applied and the guidelines for passing the exams must be made known.

In 2008, the Science Council recommended the creation of a functioning evaluation system on an international level for performance checks in universities. The task of the assessment tools applied was to analyse teaching performance clearly and dependably [http://www.wissenschaftsrat.de/download/archiv/8639-08.pdf, cited at 23.10.2015]. On the other hand, the current regulations on the licensing of dentists from 1955 contain no guidelines on the examinations held in the course of studies [http://www.gesetze-im-internet.de/z_pro/BJNR000370955.html, cited at 23.10.2015].

Because in the study of dentistry practical skills are reinforced, and thus also examined, we frequently deal with the implementation of competence-orientated methods of examination that can be characterised on the Miller pyramid by “shows how” or “acts” [4]. From this context, OSCE (Objective Structured Clinical Examination) and OSPE (Objective Structured Practical Examination) methods of examination are especially possible [4].

The OSCE method of examination was introduced in 1975 by Harden [5]. Initially conceived for examinations in medicine, today the OSCE is also used for examinations in dentistry. In a 1998 study, Mangour and Brown [6] presented the development and implementation of OSCEs in dentistry for the first time. The terms OSCE and OSPE are usually applied as equivalents and thus with no differentiation. Both Natkin and Guild [7], as well as the AMEE (Association for Medical Education in Europe) Guide No. 81 Part I [8] describe OSPE (as a variation of OSCE) as a method of examination used to test practical skills and knowledge in a non-clinical environment. The authors Wani and Dalvi [9] also noted that the OSPE is an exam form where both the strengths and weaknesses of students’ practical skill can be presented and reviewed. Students and examiners evaluate this exam form as positive and useful [1], [10], [11], [12], [13], [14]. In further studies, such as those of Smith et al. [15], Nayak et al. [16] and Abraham et al. [12], students described both OSCEs and OSPEs in comparison to written and oral examinations as fairer and less stressful exam forms, and preferred the OSPE to more “traditional” exam forms. A study by Schoonheim-Klein et al. [17] was also able to show that OSCEs, in a dental context in particular, promoted skills in the area of clinical competence and learning, as well as a more realistic self-assessment on the part of the students. In addition, the study by Nayak et al. [16] was able to show that through the OSPE, as well as the individual competencies of each student, the practical demonstration of facts and applied knowledge and learning behaviour could be positively influenced.

Reliability values between 0.11 and 0.97 were given for the OSCEs [18]. The strongly varying results can be explained primarily by the fact that the parameters under which an OSCE is held (number of stations, number of examiners, length of the exam, type of evaluation mode) could be seen to vary considerably.

Independently of the exam form, a differentiation is normally made in evaluation between the methods of “glance and grade” and evaluation based upon defined criteria. These methods were evaluated within the context of dental examination settings [19], [20], [21], [22], [23], [24], [25], [26], [27], [28], [29], [30], [31]. The majority of the studies referred to above were not able to determine any significant differences between glance and grade and criteria-based methods. Furthermore, they did not take place in a real, but rather an artificial exam environment.

There are hardly any studies on OSPEs which, as already mentioned, represent in the strict sense a variation of the OSCE on the assessment of parameters referred to above. It has not been investigated, for instance, to what extent the number of examiners and the type of evaluation methods influence the result of an OSPE.

Against this background, the aims of this study were to evaluate the reliability of a real OSPE end-of-semester exam in the phantom course of operative dentistry in Frankfurt am Main, taking various evaluation modes and number of examiners into consideration.

## Material and Methods

**Figure 1:**
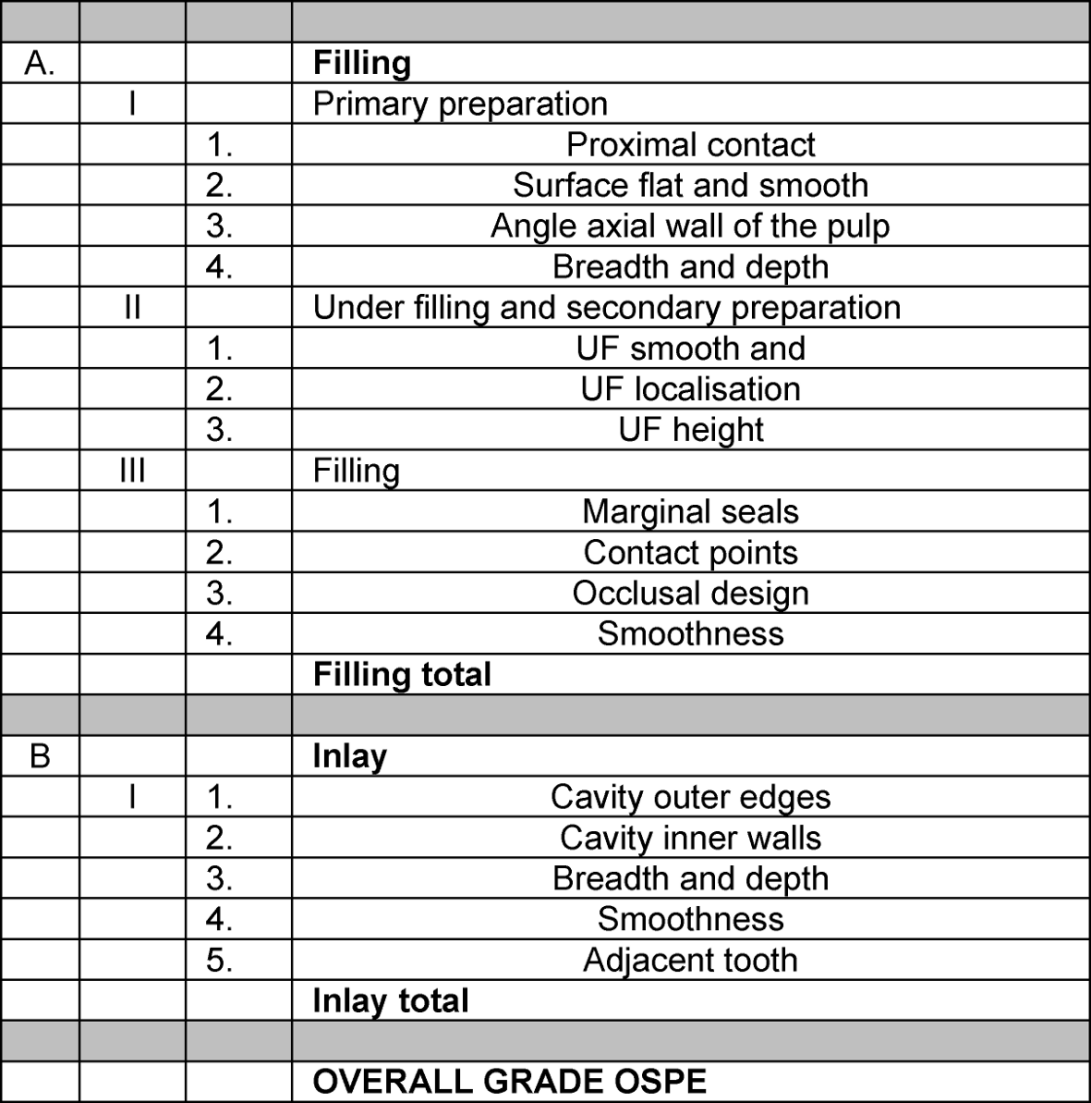
Examiner’s checklist group I and group II with both tasks A (filling) and B (inlay). The abbreviation UF stands for under filling.

The phantom course of operative dentistry ran for a period of one semester (16 weeks). During this time, students had to complete practical work on a variety of simulation models (on extracted human and industrially manufactured artificial teeth). By means of previously defined treatment protocols, various treatment alternatives (for example fillings, laboratory restorations such as inlays, endodontic treatments, etc.) were practised step by step with the help of instructors. As soon as the predefined criteria were fulfilled, each step was ratified by the supervising instructor in a so-called certification booklet. The learning process was accompanied by formative feedback. At the end of the course, both an oral test of knowledge and a summative OSPE took place. The latter was carried out in the simulation unit of so-called “phantom patients”. Two plastic models (upper and lower jaw) were mounted in a “phantom head” consisting of 14 plastic upper jaw teeth and 14 plastic lower jaw teeth. The OSPE consisted of two examination parts, the “filling” (A) and the “inlay” (B), carried out on two different plastic teeth of each respective model. These divided into six “sub-units” (1. “primary preparation”; 2. “under filling and secondary preparation”; 3. “filling”; 4. “inlay”; 5. “filling overall” and 6. “overall grade”) which were each evaluated by the examiners (see Figure 1 (Fig. 1)). These subunits accorded to the criteria based on which the attendance certificates for the course were issued by the instructors. The examiner’s checklist, which contained the list of partial aspects (subunits) mentioned above, was tested out over four consecutive semesters (summer semester 2008 to winter semester 2009) in a regular examination scenario. During the test, the evaluation took place via inspection of the prescribed partial aspects, judged purely on the basis of the view of the examiners’ general quality criteria. School grades were awarded from 1 to 5 (1=very good to 5=insufficient).

Each examiner evaluated each student in a real examination scenario (duration: 3 hrs.). This meant that the examiners assessed the students’ work directly at the workplace (on a phantom patient) in a predetermined order during the examination. The students signalled to the examiners that they were ready to submit a subunit for evaluation. During the OSPE, the examiners exchanged no information on the grades they had awarded. After the examiners had independently completed their individual examiner’s checklists, the evaluations were discussed in a joint meeting and it was determined which students should repeat the exam. This took place according to the Delphi principle [http://www.horx.com/zukunftsforschung/Docs/02-M-09-Delphi-Methode.pdf, cited at 23.10.2015].

### Examination scenario of the study

The present study relates to a period of two semesters (summer semester 2010 = Group I, summer semester 2012 = Group II). The composition of the study population is given in Table 1 (Tab. 1). The inclusion criteria were:

- students from the 6th semester
- participation in the phantom course for restorative dentistry
- examination skills present.

The exclusion criteria were defined as follows:

- students from other semesters
- course dropouts and course repeaters
- examination skills not met.

The difference in the respective group sizes (I versus II) resulted from the actual size of the semester which was subject to large variations and which was dependent upon the results of the preceding examination. A numerical adjustment of both groups was not feasible as all course participants, according to the study regulations, had to take the exam. The determination of the number of examiners was carried out prior to this study on application for ethical approval. The assignment of identical examiners for both groups was not practical for staffing reasons in the department.

**Figure 2:**
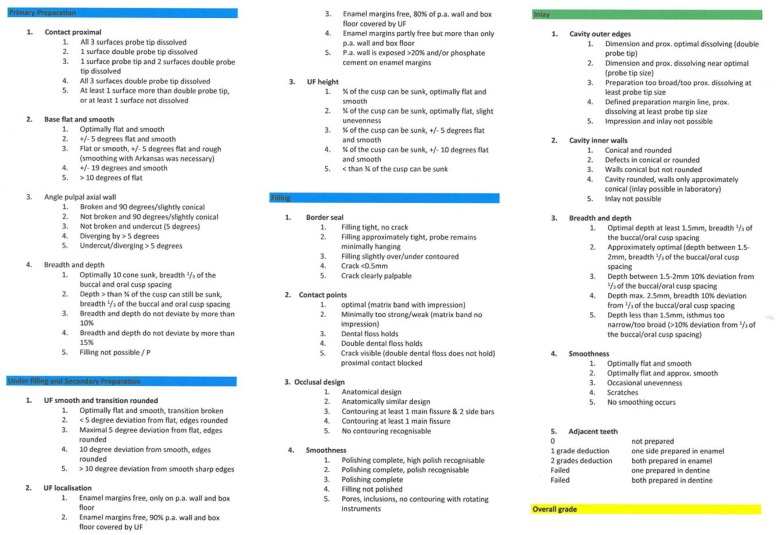
Instructor’s manual for group II with the evaluation criteria of both tasks A and B. The abbreviation p.a. signifies pulpal axial wall. The abbreviation UF stands for under filling, prox. = proximal.

In group I, an examiner’s checklist was applied exclusively, as seen in Figure 1 (Fig. 1). In group II, the examiners used the identical examiner’s checklist, but in combination with a detailed instructor’s manual (see Figure 2 (Fig. 2)). This contained clearly defined criteria for the evaluation the individual school grades.

In all, five examiners took part in the study (A-E), four women and one man. The examiners were all dentists in the Department for Operative Dentistry, had experience in teaching and in the evaluation of students’ work in the phantom course. Table 2 (Tab. 2) shows their distribution according to number and sex. Examiner A had passed the final examination in dentistry in 1990, examiners B, C, D, and E in 2007, 2008, 2010 and 2011 respectively. They all had experience in conducting the phantom course of operative dentistry. In addition to the others, only A had experience in conducting courses in patient treatment.

The examiner’s checklist originated from subject areas that were presented as standard in the current course, and in textbooks for restorative dentistry. These were also similar to the units (filling, inlay) and subunits defined as relevant for examination in operative dentistry raised in Baumann’s study [32] on an interdisciplinary basis between four centres (the universities of Frankfurt, Freiburg, Leipzig and Munich). From the manual attached to group II, examiners were able to learn which evaluation criteria had to be fulfilled in order for a particular grade to be awarded.

#### Train-the-Teacher

In each semester, a 45 minute “train-the-teacher course” was held. In this course, examiners were prepared through practical exercises and theoretical instructions on situations in the OSPE and the use of the examiner’s checklist and the instructor’s manual. Thus in advance a relatively high measure of standardisation between the examiners could be achieved.

#### Statistics and Application for Ethical Approval

**Figure 3:**
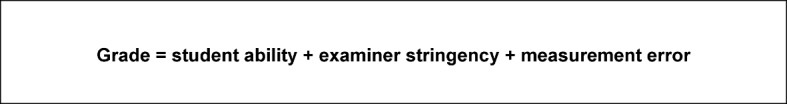
The facets of the variance analysis conducted in the study.

The results were evaluated according to the generalisability theory (G theory) with the statistic programmes SAS 9.2 (SAS Institute Inc., Cary, USA, PROC MIXED) and R (Version 2.15, Package lme4). The variance of the grades obtained is attributed to the influencing factors (in the terminology of the G theory “facets”) “students” and “examiners”, as well as to a measurement error component (see Figure 3 (Fig. 3)). From the variance proportions of the facet “examiner” and error variance relative to the facet “student”, the measurement reliability of the evaluations can be estimated. The generalisability coefficient represents an analogue to internal consistency (Cronbach’s alpha). In contrast to its usual application to various tasks, it is used here for several examiners. The G theory allows assessment of measurement reliability with the adoption of a different number of examiners to that in the actual investigation. In this way, both studies in which a varying number of examiners were involved can be made compatible (in analogy to the Spearman-Brown formula with which a standardisation of reliability for a certain number of tasks is possible).

Similarly, the individual examiners (A-E) were evaluated amongst themselves with regard to the parameter “overall grade OSPE”. A sub-group analysis taking in all parameters of examiners A and B completed the statistical analysis.

An application for ethical approval for the monocentric comparative study was given the approval number 135/35 by the Ethic Commission of the Department of Medicine of the Goethe University

## Results

Table 3 (Tab. 3) shows the results of the determination of reliability from group I using the examiner’s checklist without the instructor’s manual. In this group, only in the case of three examiners were Cronbach’s alpha values under 0.6 determined for the two criteria “interior wall of the cavity” and “breadth/depth”.

In all other subunits, the required value of 0.6 or larger than 0.6 for sufficient reliability could be attained. The subunit “adjacent tooth” achieved the value 1.0; this can be regarded as an ideal reliability value. Furthermore, table 3 (Tab. 3) shows the results of the determination of reliability from group II (using the examiner’s checklist and the instructor’s manual). In order to enable a comparison of the generalisability coefficients in both studies, these were each converted for numbers of both three and four examiners. Thus with the aid of the Spearman-Brown formula, for study I the reliability values for four examiners were determined from those for three examiners, and vice versa for group II.

In group II the results for 4 examiners showed a high variance in the calculated Cronbach’s alpha values. For the first subunit “primary preparation” and the accompanying criteria (“proximal contact point” to “breadth/depth), Cronbach’s alpha values under 0.6 were calculated. The same was the case for the subunit “filling” and the accompanying criteria “contact points”, “occlusal design” and “smoothness”, for “inlay total” and accompanying criteria such as “cavity outer edge”, “cavity inner walls”, “breadth/depth”, “smoothness” and “adjacent tooth”. The remaining subunits and criteria were able to achieve the required value for sufficient reliability of 0.6.

When comparing individual examiners regarding the parameter “overall grade OSPE”, for the summer semester 2010, correlation coefficients of 0.58 (A versus C), 0.64 (A versus B) and 0.68 (C versus B) were calculated. In the summer semester 2012, the corresponding values were lower (A versus B: 0.33; A versus E: 0.35; A versus D: 0.34; E versus D: 0.52; B versus D: 0.37 and E versus B: 0.35). The results of the subgroup analysis (A versus B, used in both study groups) can be seen in table 3 (Tab. 3).

## Discussion

### Limitations

One limitation of the present study lies in the type of trial design selected (historical comparison group), as the study was carried out not within one particular semester with a particular student population, but rather in two successive semesters with different participants. Because of two different modes of assessment, a division of the summative examination within the semester was declared inadmissible by the faculty’s ethics commission. The authors see one further limitation in the fact that the examiners from both investigated groups were not equal either in number or team composition. Only two examiners (A and B) evaluated similarly in both study groups. Furthermore, despite the preceding train-the-teacher events, a difference in teaching experience must be assumed. This variation could, however, not be homogenised for staff reasons (expiry of contracts). The elaborate statistical analysis takes account of this limitation and standardises the unequal number of examiners. 

#### Modes of evaluation

Based on current scientific information, no clear conclusion can be drawn on the benefit of an examiner’s checklist regarding the reliability of an examination. According to the latest research, there are only two studies which have dealt with the different modes of evaluation [19], [20], [26], [28], [29], [33]. In the present study, the best results could be determined regarding a high level of reliability by using the examiner’s checklist without the additional use of an instructor’s manual. A comparable result was achieved in a study by Bazan and Seale [34], where a similarly conceived examiner’s checklist for exam evaluation led to a similar reliability value for the exam. An explanation for this might be that the degree of differentiation in the evaluation guidelines was possibly too detailed to be applied by the examiner during the practical examination, and that the train-the-teacher event was apparently not able to set comparable evaluation standards for the examiners. This problem became particularly apparent in the partial step “inlay adjacent tooth” in which the extensive manual with the defined sub-criteria led to a massive deterioration in the Cronbach’s alpha values. This is also accords with the study by the authors Houpt and Cress [31], which found that the narrower the definition of the predetermined evaluation framework for a criterion was, the sooner discrepancies in measurement accuracy and examiner assessment occurred. A direct comparison of examiners A and B, who examined in both semesters, found that the use of the manual lowered the average correlation (0.68) recorded in summer semester 2010 to a value of 0.33. Despite this clarification, it is still necessary to establish why this partial step in particular caused such extreme deviations. Possibly the wording of the tooth structure definitions (enamel and dentine) resulted in confusion on the side of the examiners as the exam tasks were not carried out on natural teeth consisting of enamel and dentine, but rather on exam teeth made of plastic. Future studies should discuss the exact wording of the manual parameters in terms of content.

#### Examination setting

In contrast to the two studies already referred to, the examiners’ evaluation in the present study took place in a real exam situation. As a potential future alternative regarding study design, it would be feasible to give the examiners more time for evaluation. This, however, would require a fundamental revision of the end-of-semester exam at the University of Frankfurt am Main under study here. Considering that three hours were allowed for the whole examination, and that the individual steps were checked simultaneously ad hoc by the examiners with an average of = 22 students, more time spent on the evaluation could only be realised with difficulty. The question arises of why, during the real OSPE examination scenario, so much effort is expended and why the individual steps cannot be evaluated jointly by all the examiners after the exam. The reason for this is that many individual steps during the exam are no longer assessable owing to the succeeding phase, as they are then no longer visible. For example, the “primary preparation” step succeeding “under filling lining/secondary preparation” is no longer assessable as the former is partially concealed after putting in an under filling. This is the same for all partial steps so that at the end of the examination stage “filling”, only the final resulting step remains assessable.

This procedure stands in stark contrast to all previously published OSPE examinations where in general the individual steps were both visible and assessable, even after the examination. Compared to the studies made by Goepferd and Kerber [26], Vann et al. [28] and Scheutzel [33] there is a clear difference, as in the examinations investigated there, the similarly complex revaluation form was able to be used under more favourable time conditions. This might explain the different results between the investigation carried out here and the studies previously referred to.

#### Train-the-Teacher

OSCE-based examinations show some disadvantages by way of analogy to the advantages already referred to above. According to Miller [4], [35], experience has shown that the OSCE is particularly training intensive and time consuming, and according to Nayak et al. [16], it requires intensive planning and team work. As a rule, the appointed examiners require intensive and systematic training in order to be able to fulfil the requirements of reliability and validity for an OSCE exam [35]. As a result, the OSCE is time consuming and cost intensive in comparison to other exam types such as multiple choice or oral exams [8], [35], [36]. In the context of the present study, a time-consuming preparation of the examiners in a train-the-teacher event was also carried out. As a result, resources of personnel and space, as well as financial resources in the clinical and organisational workflow within the department for restorative dentistry, would have to be found. The duration of a lecture unit (45 mins.) was realistic for this purpose and could be observed by all the examiners. However, the question arises as to how long preparation should effectively be in order to be able to homogenise different experiences in mixed teams in advance. In the summer semester of 2010, the three examiners amongst themselves showed an average correlation of between 0.58 and 0.68. In the summer semester of 2012, in the case of four examiners the identically long train-the-teacher events resulted in correlation values of 0.33 and 0.52. It can be assumed here that in the case of the application of the manual, the train-the-teacher event was not effectively utilised.

#### Examiners 

On the basis of current data, examiners play an important role in the assessment of reliability. Until now, however, there have been no scientific studies known to us that have made any assessment of how high the minimum number of examiners for a OSPE should be. In this study, it was possible to attain sufficient reliability with three examiners in combination with checklists. According to the results of this investigation, the reliability value can be increased by a higher number of examiners. This increase in reliability values, however, is low in comparison to the number of examiners. In addition, a further increase in the number of examiners would result in greater complexity and expense with regard to organisation and financial costs.

In this context, it has to be mentioned critically that no general recommendation can be made for other sites based upon the data available with regard to the number of examiners, as the possibility of having three to four examiners with long experience available for an OSPE examination is neither representative of normal circumstances nor feasible. The author groups Nikendei and Jünger [37] and Norcini et al. [38] came to a similar result. In their study, Natkin and Guild [39] were able to show a significant increase in reliability through a systematic preparation of the evaluators. Similar results were presented by Dhuru [25], in whose study examiners with many years of professional experience and using evaluation sheets achieved the most reliable examination results. In the present study, this can be confirmed only with the use of the checklist, as when the manual was used, the two examiners with the most years’ experience demonstrated only weak correlations. As shown in this investigation, the checklist appears to be capable of further increasing reliability, or of compensating for a lack of examining experience on the part of the evaluators. In Houpt and Kress’s [31] investigation, by contrast, reliability could not be increased for all evaluation criteria. Thus the authors believe that the train-the-teacher events on their own are not able to increase interrater reliability significantly. Training events of this type had the greatest effect with “non-expert” examiners, but relatively little influence with experienced evaluators [31]. Our study was able to confirm this. 

#### Exam tasks

The number of examination tasks defined in this study, frequently equated with the term “stations” in the literature, should be looked at critically. In the present case only two separate tasks were involved (A. filling and B. inlay), but a total of 22 evaluations were obtained by the evaluators per student in and during the exam. Ultimately we are dealing with the definition of the term “station” in connection with the OSPE which based upon the evidence cannot be deduced from the literature. It must be noted critically that a value of 0.6 for Cronbach’s alpha only has a “sufficient” character. It must therefore also be asked just how valid an examination can then be, and whether it is suitable as a summative examination. According current scientific knowledge, it is our opinion that against this background, variant II cannot be recommend for high stakes examinations.

## Conclusion

The following conclusions may be drawn from this study regarding the question of how an OSPE in dental teaching in a phantom course for operative dentistry can best be reliably designed:

- an examiner’s checklist without an instructor’s manual resulted in higher interrater reliability in the context of the OSPEs carried out
- the evaluation of students’ exam performance in the context of the OSPE should if possible be undertaken by at least three examiners.

## Acknowledgements

The authors would like to thank the students of the 6th semester in the section for operative dentistry and the dental course assistants who also contributed to the evaluation of the OSPE.

## Competing interests

The authors declare that they have no competing interests.

## Figures and Tables

**Table 1:**
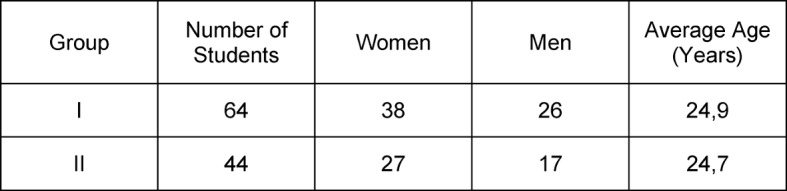
Composition of the study population.

**Table 2:**
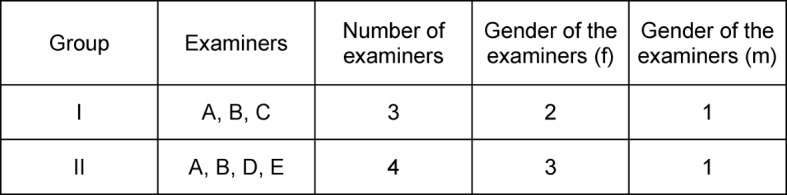
Data for the examiners A to E, who evaluated group 1 in SS 2010 (A, B, C) and group 2 in SS 2012 (A, B, D, E) (f = female, m = male).

**Table 3:**
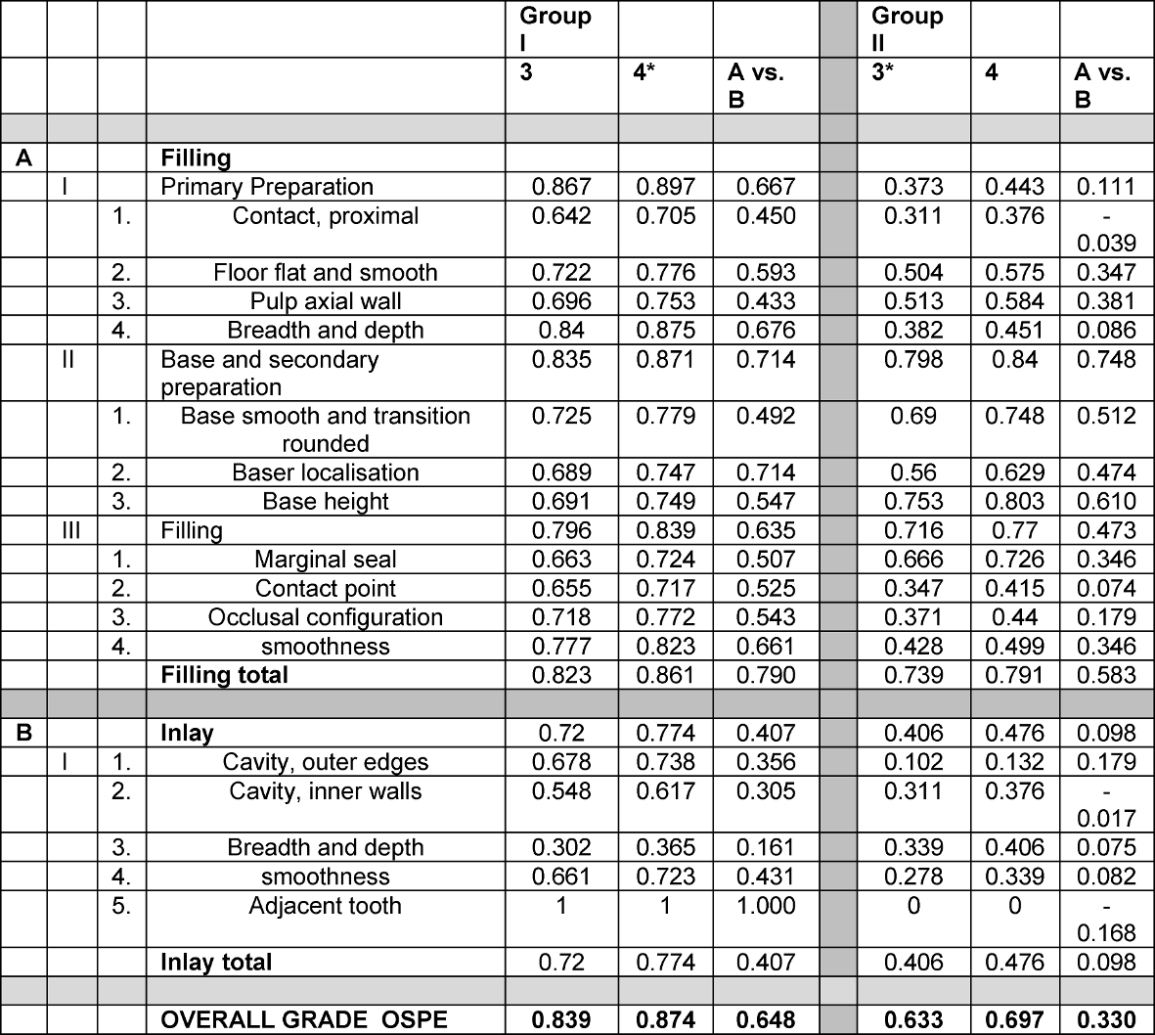
Results of group I and group II. The corresponding reliability values (Cronbach’s alpha) are given for three and four examiners. In the column “A vs. B”, the results of the subgroup analysis are presented. Identification with * means that differing from the real examination scenario, a conversion into another number of examiners (abbreviations: CL = cavity lining, vs. = versus).
